# Socio-ecological factors linked with changes in adults’ dietary intake in Los Angeles County during the peak of the coronavirus 2019 pandemic

**DOI:** 10.1017/S1368980024001034

**Published:** 2024-05-07

**Authors:** Sydney Miller, Trevor A Pickering, Wändi Bruine de Bruin, Thomas W. Valente, John P Wilson, Kayla de la Haye

**Affiliations:** 1 Keck School of Medicine, University of Southern California, Los Angeles, CA 90032, USA; 2 Dornsife School of Public Health, Drexel University, Philadelphia, PA 19104, USA; 3 Center for Economic and Social Research, Dornsife College of Letters, Arts and Sciences, University of Southern California, Los Angeles, CA, USA; 4 Sol Price School of Public Policy and Dornsife Department of Psychology, University of Southern California, Los Angeles, CA, USA; 5 Schaeffer Center for Health Policy and Economics, University for Southern California, Los Angeles, CA, USA; 6 Spatial Sciences Institute, Dornsife College of Letters, Arts and Sciences, University of Southern California, Los Angeles, CA, USA; 7 Viterbi School of Engineering and the School of Architecture, University of Southern California, Los Angeles, CA, USA

**Keywords:** Socio-ecological factors, Dietary patterns, COVID-19, Social networks, Neighbourhood environments

## Abstract

**Objective:**

Comprehensive studies examining longitudinal predictors of dietary change during the coronavirus disease 2019 pandemic are lacking. Based on an ecological framework, this study used longitudinal data to test if individual, social and environmental factors predicted change in dietary intake during the peak of the coronavirus 2019 pandemic in Los Angeles County and examined interactions among the multilevel predictors.

**Design:**

We analysed two survey waves (e.g. baseline and follow-up) of the Understanding America Study, administered online to the same participants 3 months apart. The surveys assessed dietary intake and individual, social, and neighbourhood factors potentially associated with diet. Lagged multilevel regression models were used to predict change from baseline to follow-up in daily servings of fruits, vegetables and sugar-sweetened beverages.

**Setting:**

Data were collected in October 2020 and January 2021, during the peak of the coronavirus disease 2019 pandemic in Los Angeles County.

**Participants:**

903 adults representative of Los Angeles County households.

**Results:**

Individuals who had depression and less education or who identified as non-Hispanic Black or Hispanic reported unhealthy dietary changes over the study period. Individuals with smaller social networks, especially low-income individuals with smaller networks, also reported unhealthy dietary changes. After accounting for individual and social factors, neighbourhood factors were generally not associated with dietary change.

**Conclusions:**

Given poor diets are a leading cause of death in the USA, addressing ecological risk factors that put some segments of the community at risk for unhealthy dietary changes during a crisis should be a priority for health interventions and policy.

Most individuals in the USA do not adhere to national dietary recommendations^(1)^, especially with regard to fruit, vegetables and added sugar. Only 12·2 % of adults meet recommendations for fruit intake, and 9·3 % meet recommendations for vegetable intake^(2)^. Most adults also exceed recommendations for added sugar, and though it is recommended to avoid all sugar-sweetened beverages (SSB) due to their high sugar content^(1)^, one-half of USA adults consume at least one SSB per day^(3)^. As such, poor dietary patterns are a leading cause of disease and excess death in the USA^(4)^, and there are also extensive socio-economic and racial and ethnic disparities in diet-related diseases^(2,5)^.

The coronavirus 2019 (COVID-19) pandemic substantially changed the diets of some Americans – with diet quality improving for some and declining for others^(6–8)^. Specifically, studies have shown decreases in the consumption of fast food but increases in the consumption of SSB and ultra-processed foods^(6–8)^. Additionally, studies have documented a mix of both increased and decreased consumption of fruits and/or vegetables^(6–8)^. However, the factors causing these changes are not well understood. Some of the key limitations of this research are that it has been cross-sectional and has sought to describe the magnitude of dietary shifts, with less emphasis on identifying contributing factors^(6–8)^.

The pervasiveness of poor diets is argued to be ‘not a problem of knowing, but a problem of doing^(9)^’, with barriers to healthy eating occurring across many contexts^(10)^. Adopting an ecological framework^(10)^, it has been posited that there are key individual, social and neighbourhood-level factors that *independently* and *interactively* influence diet. Many of these factors were substantially disrupted during the most acute phase of the COVID-19 pandemic^(11–13)^. For example, social-distancing policies and the widespread closures of businesses and schools led to increases in financial and food insecurity, weakening of social networks and changes in neighbourhood social and food environments^(11,14,15)^. However, among the few studies in the USA that sought to identify predictors of dietary change during the pandemic, most focused on individual-level factors^(16–18)^. These studies found that financial stress and food insecurity were linked to decreases in fruit and vegetable consumption^(17,18)^, while identifying as non-Hispanic Black or Hispanic, or having lower education or income, was linked with increases in SSB consumption^(19,20)^. We identified one cross-sectional study that examined broader social or neighbourhood factors by utilizing a retrospective question on perceived dietary change^(16)^. This study found that individuals were more likely to report healthy changes if they identified as non-Hispanic Black or Hispanic/Latino, had received COVID-19 financial assistance, or had larger social networks, while individuals were more likely to report unhealthy changes if they were younger or had transportation barriers to accessing food^(16)^.

Given the research gaps above, longitudinal studies are needed to examine the independent and interactive associations of key individual, social and neighbourhood-level factors with changes in dietary intake during the peak of the COVID-19 pandemic in Los Angeles (LA) County, to inform comprehensive, multilevel intervention and policy strategies. In alignment with the ecological framework^(10)^, we utilised longitudinal data to examine whether key multilevel factors that have historically predicted diet and that have also been substantially impacted by the COVID-19 pandemic^(11–13)^ predicted changes in diet during this period (Aim 1). We also examined if these influences were interactive (e.g. if risk factors at multiple levels of influence were associated with more negative changes in dietary intake) (Aim 2).

## Methods

### Ecological framework

This study uses an ecological framework, which posits that there are multilevel influences on dietary patterns that can be organised into individual, social, neighbourhood and policy-level influences^(10)^. This framework was adopted because it has been used to articulate and identify specific influences on dietary intake, and on the intervention intervention and policy implications of these multilevel relationships. Additionally, this framework was selected because it emphasises multilevel factors as part of a complex, interdependent system, where the effect of factors across levels is often *synergistic and interactive* in nature^(10)^. For example, the negative effects of living in poverty (an individual barrier) on the capacity to eat a healthy diet might be worsened by having few social connections who can provide social support (a social barrier) or living in a neighbourhood with limited healthy food access (a neighbourhood barrier). Based on this framework, we identified multilevel factors that have historically predicted diet and have been altered substantially during the pandemic (described below in Measures)^(11–13)^, and we test for both main and interaction effects.

### Los Angeles County

This study focuses on LA County, an area that was substantially impacted by the COVID-19 pandemic. LA County has had more than 3·6 million documented COVID-19 cases among its population of 10 million. During the peak in January of 2021, there were an average of 200 deaths per day^(21)^. Due to a high rate of cases and deaths, social-distancing policies were expansive and long-standing, as were business and school closures^(11,12,21)^. Thus, daily lives were disrupted in LA County, with substantial shifts in individual, social and structural factors potentially linked to dietary intake. The broad socio-demographic diversity of the LA County population and geographic landscape provides sufficient variation for these aims.

### Participants and procedures

Data were from the Understanding America Study (UAS)^(22)^, a probability-based online panel of adult USA residents (18+ years old) that began in 2014. The UAS collects regular surveys among panel participants covering a variety of topics related to demography, health and ageing. Participants complete surveys online, in English or Spanish. They are provided with a tablet and Internet access if needed. Participants are compensated for their participation based on survey length.

The UAS includes a subsample representative of LA County, recruited from randomly selected county addresses with sampling probabilities adjusted for underrepresented populations. Post-stratification weights, developed for each survey wave, are used to further align the sample to LA County’s adult population regarding age, gender, race/ethnicity and education. The UAS was approved by the Institutional Review Board of the University of Southern California.

Beginning in March 2020, the UAS started high-frequency longitudinal surveys about the COVID-19 pandemic (the ‘Understanding Coronavirus in America Tracking Survey’). This study focused on measures assessed at two survey waves, in October 2020 (baseline) and January 2021 (follow-up). January 2021 was the peak of the pandemic in LA County, when there were the most hospitalisations and deaths than in any other period from 2020 to 2023^(21)^. Panel participants who did not complete baseline *and* follow-up surveys were excluded from the analytic sample, yielding a final sample size of 903 individuals who provided data at both baseline and follow-up (data were matched by participants across both waves). χ^2^ tests were used to compare characteristics of the full UAS LA County subsample with the analytic sample, and the two samples did not differ significantly on basic demographics (gender, age, income, education, and race and ethnicity). All descriptive statistics and analyses were computed using survey weights, so that the results remain representative of LA County, even with the missing participants.

### Measures

#### Outcomes

##### Dietary intake

Dietary intake was assessed at baseline and follow-up. Dietary intake was self-reported using validated questions from the California Health Interview Survey and assessed intake of vegetables, fruits and SSB as these are key food types that are important to diet quality and nutritional health^(23)^. For all three food types, respondents entered a number of servings and then selected from the following referent time periods: per day, per week or per month.

##### Vegetable intake

Vegetable intake was measured by the question: *During the past month, how many times did you eat vegetables like green salad, green beans or potatoes? Do not include fried potatoes or cooked dried beans such as refried beans, baked beans or bean soup. Other vegetables include tomatoes, carrots, onions or broccoli. Rice is not a vegetable. You can indicate if this is per day, per week or in a month.*

##### Fruit intake

Fruit intake was measured by the question: *During the past month, how many times did you eat fruit? Do not count juices. You can indicate if this is per day, per week or in a month.*

##### Sugar-sweetened beverage (SSB) intake

SSB intake was measured by the question: *During the past month, how often did you drink sodas or sweetened fruit drinks, sports or energy drinks? Do not include diet sodas or sugar-free drinks. Please count a 12-ounce can, bottle or glass as one drink. Examples might include sweet lemonade, Coke, Gatorade, Snapple or Red Bull. Do not include 100 % fruit juices, yogurt drinks, carbonated water or fruit-flavoured teas. You can indicate if this is per day, per week or in a month.*

These responses were used to compute daily servings of each food type, by dividing servings reported in weekly or monthly units by 7 d or 30 d, respectively. Servings of fruits and vegetables were analysed separately because the COVID-19 pandemic differentially affected the intake of fruits *v*. vegetables (i.e. some studies documenting increases in fruit intake but decreases in vegetable intake)^(6–8)^.

#### Predictors and covariates

All predictors and control variables were measured at baseline (October 2020), unless otherwise noted.

##### Individual predictors

Decreases in mental health have been a key concern of the COVID-19 pandemic^(17,24)^. Depression and anxiety were assessed using the validated four-item Patient Health Questionnaire^(25)^, which assesses feelings of depression and anxiety over the past 2 weeks. Participants were asked, *Over the last 2 weeks, how often have you been bothered by the following problems?: (i) Feeling nervous, anxious or on edge; (ii) not being able to stop or control worrying; (iii) feeling down, depressed or hopeless; and (iv) little interest or pleasure in doing things.* Response options were not at all, several days, more than half of days or nearly every day, scored 0, 1, 2 or 3, respectively. The scale was designed to be scored into two binary variables: (i) scores of ≥ 3 on the depression subscale (items 3 and 4) were designated as reflecting depression (yes = 1/no = 0), and (ii) scores of ≥ 3 on the anxiety subscale (items 1 and 2) were designated as reflecting anxiety (yes = 1/no = 0).

Given the financial and logistical complications that the COVID-19 pandemic has created in accessing food and the emergency expansion of food assistance programmes in California^(11)^, we also examined the receipt of food assistance. This was assessed by using a standard UAS question to ask respondents if, in the past 2 weeks, any person in their household had received Supplemental Nutrition Assistance Program benefits or Women, Infants, and Children benefits. Households who received either were categorised as receiving food assistance (yes = 1/no = 0).

Past-month food insecurity was measured using the validated Food Insecurity Experience Scale^(26)^. This scale assesses food insecurity over the past 7 d: (i) *Did you eat less than you thought you should because of a lack of money or other resources?* and (ii) *Did you go without eating for a whole day because of a lack of money or other resources?* (yes = 1, no = 0). These questions assessed behavioural markers of moderate and severe levels of food insecurity, respectively^(26)^. Participants who responded yes to at least one question were considered to have experienced past-week food insecurity in that wave. Participants were categorised as experiencing any food insecurity if they indicated food insecurity during any week over the previous month. Given the vast increases in food insecurity and the relationship with dietary intake during the pandemic in LA County^(16)^, food insecurity was retained in all of the models.

##### Social network predictors

Social networks (i.e. the family, friends and other individuals whom one is connected to) may play a role in acquiring adequate finances, food and other resources that impact diet^(27)^, and social networks were heavily disrupted during the pandemic^(14,15)^. We examined (i) self-reported social network size and (ii) self-reported social network members that provided food-related support (e.g. social supporters). These social network questions have been used in the UAS historically and have been found to be important predictors of voting behaviour, vaccination, disease screening and other outcomes^(28,29)^. Social network size was assessed in July 2020 (2–3 months prior to ‘baseline’) with the question, ‘About how many friends and family members do you have?’ As a check, all participants were then asked, ‘Are you sure you really have ___ friends and family members?’ Following this question, the number of social supporters was assessed by the question, ‘In the past 30 d, how many of these family and friends helped you to get enough food to eat, by sharing money, resources or food with you?’ Social network size was highly skewed, and several versions of this variable were explored (e.g. log-transformed continuous, categorisation by quartiles, binary categorisation). Results did not differ based on these different versions, so a binary categorisation was chosen for simplicity in interpreting interaction results. This binary categorisation was based on the median value of sixteen network members (below the median of 16 *v*. median of 16 or above). For social network supporters, 72·1 % of respondents indicated they had zero social supporters; thus, this variable was also categorised into a binary predictor (no supporters *v*. one or more supporters).

##### Neighbourhood environment predictors

All neighbourhood environment measures were captured at the census tract level. Validated measures from multiple secondary data sources were obtained as described below.

Neighbourhood food environments have been linked with diet^(30–32)^, but they may have been even more impactful during the pandemic, as individuals who were more confined to their neighbourhoods were likely shopping for food close to home^(33)^. We examine indicators of both food deserts (e.g. areas where healthy food outlets are limited^(30–32)^) and overall retail food environment quality. Food desert indicators were obtained from the United States Department of Agriculture (USDA) 2020 Food Access Research Atlas^(34)^, which uses data from 2019 business listings and census. This study used the USDA-computed indicator for ‘low-income and low access’ food deserts. This USDA data was used to code the census tracts that correspond to each participant’s home address as a food desert (0 = no, 1 = yes). Retail food environment quality was captured using the modified Retail Food Environment Index (mRFEI), a measure computed and made publicly available by the California Department of Public Health using data from 2017^(35)^. The mRFEI is calculated as the percent of healthy food outlets (e.g. supermarkets) to the number of total food outlets (i.e. healthy plus less healthy food outlets) within a given census tract. Scores of zero indicate a census tract is a food desert (i.e. there is zero healthy food outlet within the census tract). Among mRFEI scores greater than zero, lower scores indicate worse quality (i.e. there is a low ratio of healthy food outlets compared with all types of food outlets), while higher scores indicate better retail quality. The mRFEI is traditionally on a scale of 0–100, but due to the Understanding America Study policies on merging spatial data to preserve participant privacy, the mRFEI was divided by 10 and rounded to the nearest half number (e.g. 0, 0·5, 1, 1·5, etc.). Any value above zero was rounded up to 0·5 so that zeros are true zeros (e.g. a food desert).

Neighbourhood social vulnerability captures a broader set of resources and social determinants of health available in one’s neighbourhood^(36,37)^. We use the Center for Disease Control and Prevention’s social vulnerability index, which captures communities’ vulnerability to the potential negative effects caused by external stresses on human health (e.g. natural disasters, disease outbreaks)^(38)^. The social vulnerability index ranks each census tract on sixteen social factors, including poverty, lack of vehicle access and crowded housing, and gives the census tract an overall ranking relative to the other census tracts in the USA. Based on the Center for Disease Control and Prevention’s social vulnerability index categorisation procedures^(38)^, census tracts with a ranking of 0·75 or higher are designated as having high social vulnerability.

##### Control variables

Standard control variables were measured, including gender (0 = male, 1 = female), education (0 = high school degree or less, 1 = some college or technical training/associate’s degree, 2 = bachelor’s degree or more), age (continuous variable) and race and ethnicity (0 = non-Hispanic White, 1 = Hispanic, 2 = non-Hispanic Black, 3 = non-Hispanic Asian, 4 = Native American, Pacific Islander, Alaska Native, Other). Respondents reported the income of their entire household over the past year and the number of individuals living in their household. This was used to compute federal poverty level and identify individuals with low income (1 = <=300 % federal poverty level, 0 = higher income, > 300 % federal poverty level, based on thresholds established by the LA County Department of Public Health (e.g. they define low-income households as those < 300 % of the federal poverty level, noting that in an area with such high cost of living, these households are higher risk for food insecurity and encompass many households eligible for government food assistance).

### Analyses

Weighted means and sd were computed for all continuous predictors and the continuous outcomes, and weighted frequencies and percentages were computed for all categorical predictors (Tables 1 and 2). Initial exploratory analysis was used to understand the distributions of all variables. Daily servings of fruit, vegetables and SSB were highly skewed and thus were log-transformed to better fit modelling assumptions.

**Table 1:** Weighted descriptive statistics for predictors and covariates for Los Angeles County adults during October 2020 (baseline; weighted *n* 898)

Variable	Frequency/mean	Percent/sd	Min-max
Individual-level factors			
Gender			
Female	450	50·1	
Male	448	49·9	
Age			
18–30 years old	167	18·7	
31–40 years old	207	23·2	
41–50 years old	137	15·4	
51–64 years old	215	24·0	
65+ years old	168	18·8	
Education			
High school/GED or less	371	41·4	
Some college	212	23·6	
Bachelors or more	314	35·0	
Race and ethnicity			
Non-Hispanic White	254	28·3	
Non-Hispanic Black	73	8·1	
Hispanic, any race	422	47·0	
Non-Hispanic Asian	135	15·0	
Other^*^	14	1·5	
Low income (yes)	494	57·8	
Past-month food insecurity (yes)	122	13·6	
Food assistance (yes)	137	15·4	
Mental health			
Depression	108	13·5	
Anxiety	113	14·1	
Social-level factors			
Social supporters (one or more)	231	27·9	
Social network size			
Quartile 1 (0–8 members)	218	24·3	
Quartile 2 (9–16 members)	238	26·6	
Quartile 3 (17–30 members)	219	24·5	
Quartile 4 (30+ members)	219	24·5	
Social network size (continuous)	26·14	26·10	0·0–100·0
Neighbourhood-level factors			
Food desert (yes)	280	31·5	
Neighbourhood social vulnerability (high)	386	43·4	
MRFEI score (continuous)	2·24	1·11	0·0–5·0

MRFEI, modified Retail Food Environment Index.

* American Indian/Alaska Native or Hawaiian/Pacific Islander.

**Table 2:** Dietary patterns in October 2020 (baseline) and January 2021 (follow-up) and longitudinal changes

Variable	Mean	sd	Min-max
Baseline			
Vegetable intake (servings per d)	1·23	1·20	0·0–8·0
Fruit intake (servings per d)	1·04	1·11	0·0–7·0
SSB intake (servings per d)	0·48	0·94	0·0–7·0
Follow-up			
Vegetable intake (servings per d)	1·19	1·03	0·0–7·0
Fruit intake (servings per d)	1·01	1·01	0·0–7·0
SSB intake (servings per d)	0·43	0·80	0·0–8·0
Variable	Frequency	Percent	
Change from baseline to follow-up^*^			
Vegetable intake			
No change	500·0	55·7	
Decrease in daily servings	198·8	22·2	
Increase in daily servings	198·2	22·1	
Fruit intake			
No change	510·9	57·0	
Decrease in daily servings	190·7	21·3	
Increase in daily servings	195·4	21·8	
SSB intake			
No change	690·2	77·3	
Decrease in daily servings	101·4	11·4	
Increase in daily servings	101·1	11·3	

SSB, sugar-sweetened beverage.

* Change of 0·5 servings or more.

A separate linear regression model was used for each of the following outcomes: log-transformed number of daily servings of vegetables, log-transformed number of daily servings of fruits and log-transformed number of daily servings of SSB. Participants were nested within census tracts; thus, all census tract level variables were level 2 variables. As there were only two time points in this study, nesting time points within each individual were not necessary; thus, all other variables were level 1 variables. All statistical tests were performed using SAS v9.4.

Pairwise tests (e.g. Pearson’s R) were used to initially explore relationships between pairs of all potential predictors and outcomes. Next, lagged regression models were used to predict the dietary intake of each food type at follow-up, controlling for intake of that food type at baseline. A stepwise approach was used to build regression models for each of the three outcomes, where control variables were used in the initial model, and then all predictors were added into the model in related groups (e.g. all social-level predictors, neighbourhood-level predictors, etc.) and retained in the next model when marginally significant (*P* < 0·10). Collinear predictors (e.g. food deserts, retail food environment quality, neighbourhood social vulnerability) were added to the models separately. Predictors were retained in the final model when they were statistically significant (*P* < 0·05) and significantly improved the fit of the model (e.g. the log likelihood, AIC, BIC). Building on these final main effects models, potential interactions between predictors were also explored and retained when they improved the fit of the model. Interactions were further explored using scatter plots and estimation of marginal effects. Finally, model diagnostics were examined to explore influential observations and evaluate the appropriateness of the final model. Residuals fit the assumptions of linearity and homoscedasticity.

## Results

### Descriptive statistics

Weighted descriptive statistics for all covariates are summarised in Table 1, and descriptive statistics for dietary outcomes are summarised in Table 2. At both baseline and follow-up, participants consumed an average of about 1 serving of fruit per day, 1 serving of vegetables and half a serving of SSB.

From baseline to follow-up, 21·3 % (*n* 191) of individuals *decreased* their fruit intake by half a serving or more, and 21·8 % (*n* 195) of individuals *increased* their fruit intake by half a serving or more. Of the 191 individuals who decreased their fruit intake, 19 % (*n* 36) decreased their intake by 2 or more servings. Of the 195 individuals who increased their fruit intake, 20·1 % (*n* 39) increased their intake by 2 or more servings.

From baseline to follow-up, 22·2 % (*n* 199) of individuals *decreased* their vegetable intake by half a serving or more, and 22·1 % (*n* 198) *increased* their vegetable intake by half a serving or more. Of the 199 individuals who decreased their vegetable intake, 27·5 % (*n* 55) decreased their intake by 2 or more servings. Of the 198 individuals who increased their vegetable intake, 17·4 % (*n* 34) increased their intake by 2 or more servings.

From baseline to follow-up, 11·4 % (*n* 102) of individuals *decreased* their SSB intake by half a serving or more, and 11·3 % (*n* 101) *increased* their SSB intake by half a serving or more. Of the 102 individuals who decreased their SSB intake, 19·3 % (*n* 20) decreased their intake by 2 or more servings. Of the 101 individuals who increased their SSB intake, 18·3 % (*n* 19) increased their intake by 2 or more servings.

### Regression model results

Results for the regression models that predicted change in dietary intake are summarised in Table 3. The final main effects models are designated as Model 1, and the final models with interactions are designated as Model 2.

**Table 3:** Predictors of change in dietary intake among Los Angeles County adults from October 2020 (baseline) to January 2021 (follow-up)

	Vegetable		Fruit	SSB
	Model 1	Model 2	Model 1^†^	Model 1^†^
Intercept	0·65^**^	0·62^***^	0·35^***^	0·10^*^
Control variables				
Female	− 0·01	− 0·01	0·00	− 0·02
Age (10 years)	0·00	0·00	0·02^**^	0·00
Low income (yes)	0·03	0·04	− 0·05	− 0·02
Education (ref: bachelor’s degree or more)				
High school, GED or less	− 0·10^*^	− 0·09^*^	0·04	0·12^***^
Some college	− 0·04	− 0·04	− 0·06	0·12^***^
Race and ethnicity (ref: non-Hispanic White)				
Non-Hispanic Black	− 0·02	− 0·02	− 0·02	0·19^***^
Hispanic	− 0·05	− 0·05	− 0·06	0·08^**^
Asian	0·01	0·01	− 0·06	0·04
Other	− 0·02	− 0·02	− 0·02	− 0·01
Food insecure (yes)	− 0·05	− 0·05	− 0·02	− 0·07^*^
Baseline intake	0·11^***^	0·11^***^	0·16^***^	0·14^***^
Individual-level factors				
Depression (yes)	− 0·12^**^	− 0·11^**^		
Social-level factors				
Social network size (below median)	− 0·10^***^	− 0·03	− 0·07^*^	
Cross-level interactions				
Low income (yes)* network size (below median)		− 0·14^*^		

SSB, sugar-sweetened beverage.

Model 1: main effects model; Model 2: interaction model.

* *P* < 0·05.

** *P* < 0·01.

*** *P* < 0·001.

† No significant interactions found for fruit intake or SSB intake.

#### Aim 1: Will multilevel factors predict dietary changes during the pandemic?

##### Vegetable intake

Compared with individuals with a bachelor’s degree or more, individuals with a high school degree or less decreased their daily servings of vegetables by 9·0 % (95 % CI –16·3, –8·6; *P* < 0·05). Compared with individuals without depression, individuals with depression decreased their daily servings of vegetables by 10·6 % (95 % CI –17·9, –10·1; *P* < 0·01).

##### Fruit intake

For every 10-year increase in age, individuals increased their daily servings of fruit by 2·6 % (95 % CI 0·8, 2·6; *P* < 0·01). Individuals who self-reported a smaller social network decreased their daily servings of fruit by 6·6 % (95 % CI –11·6, –6·3; *P* < 0·05).

##### SSB intake

Compared with individuals with a bachelor’s degree or higher, individuals with a high school degree or less increased their daily servings of SSB by 12·6 % (95 % CI 6·3, 13·5; *P* < 0·001), and those with some college or less increased their daily servings of SSB by 13·2 % (95 % CI 7·5, 14·1; *P* < 0·001). Compared with individuals who identified as non-Hispanic White, individuals who identified as non-Hispanic Black increased their daily servings of SSB by 20·6 % (95 % CI 11·3, 22·9; *P* < 0·001), and individuals who identified as Hispanic increased their daily servings of SSB by 8·0 % (95 % CI 2·2, 8·4; *P* < 0·01). Compared with individuals who self-reported being food secure, individuals who self-reported being food insecure decreased daily servings of SSB by 6·3 % (95 % CI –12·1, –6·1; *P* < 0·05).

#### Aim 2: Will multilevel factors interact to predict dietary change?

##### Vegetable intake

There was a significant interaction between income and network size ((exp(*b*)–1) × 100 = –13·1; 95 % CI –22·8, –12·2; *P* = 0·02). For individuals with low income, self-reporting a smaller social network (e.g. less than the median number of sixteen people) was associated with a 15·8 % decrease in daily servings of vegetables (*P* < 0·0001), while network size was not related with vegetable consumption for individuals with high income.

There were no significant cross-level interactions that predicted a change in fruit or SSB intake.

## Discussion

This study examined if multilevel ecological factors predicted a change in the dietary patterns of adults living in LA County during the peak of the COVID-19 pandemic. In line with the ecological framework,^(10)^ interactive factors at the individual and social levels predicted changes in dietary intake. LA County adults at risk for poor dietary changes included individuals who were younger adults, identified as non-Hispanic Black or Hispanic, had less education, experienced depression, had smaller social networks or were food secure. Individuals with low income and social risk factors had compounded risk. Some of these segments of the community have historically faced disparities in diet and related diseases^(2,5)^, resulting from social determinants of health, suggesting that diet-related disparities may have been exacerbated during the COVID-19 pandemic.

There were several individual-level factors that predicted a change in diet. Notably, individuals with depression significantly decreased their vegetable intake, adding to the growing body of evidence that widespread health initiatives that holistically target both mental and physical health in the aftermath of the COVID-19 pandemic are needed^(11,16,39)^. Unexpectedly, having low income was not a predictor of changes in dietary intake after accounting for other individual and social-level factors (though it was predictive in initial preliminary models). Rather, having less than a bachelor’s degree, or identifying as non-Hispanic Black or Hispanic was more strongly linked with negative dietary changes. Though, historically, much research has established the importance of income in determining risk for poor dietary intake, this finding aligns with other studies that have shown that other socio-demographic factors such as race and ethnicity and education may have been more important predictors of dietary change during the COVID-19 pandemic^(16)^. This adds further complexity to the conversation surrounding educational, racial, ethnic and income disparities in health, as they are all closely intertwined^(40)^.

At the social network level, social network size was predictive of change in fruit consumption. From baseline to follow-up, individuals who self-reported a smaller social network decreased their consumption of fruits by 6·6 %. Larger social networks are important because they foster more social capital by offering more frequent and diverse opportunities to be connected to adequate financial, social and physical resources, especially during times of crises^(27)^. As such, these findings are in line with relevant theories and studies that suggest an association between smaller social networks and adverse health outcomes^(41,42)^.

At the neighbourhood level, after controlling for individual-level and social network predictors, neither food environments nor social vulnerability was associated with changes in dietary intake. Though we identified no studies that examined neighbourhood social vulnerability and diet during the pandemic, these findings contradict a few studies showing that higher neighbourhood social vulnerability is linked with increased risk for diet-related diseases^(36,37)^. With regard to the food environment, other studies have suggested that the mixed findings on the association between neighbourhood food environments and diet may potentially be due to confounding factors like neighbourhood socio-economic status^(43)^. Overall, the results of this study align with the literature suggesting that the relationships between food environments and dietary intake are complex and that other factors should also be considered, such as other neighbourhood-level indicators, as well as food price and personal food preferences^(30–32)^.

A second aim of the study was to explore interactions between multilevel predictors of dietary change, and we found that for low-income individuals, reporting a smaller social network was linked with a 15·8 % decrease in vegetable consumption. This was a considerable decrease, given the average intake of only one serving of vegetables per day. Notably, social network size was not linked with changes in vegetable intake for high-income individuals. As a larger social network size is indicative of more access to social capital and resources such as money, food and job opportunities^(27,44,45)^, these social resources may be much less meaningful for higher-income individuals who are already well-resourced. On the other hand, for low-income individuals, social capital can improve access to much-needed resources and buffer against other multilevel barriers to health^(44,45)^. This further points to the growing body of evidence that emphasises the importance of considering the social contexts surrounding individuals with low income^(10)^.

### Strengths

This study is among the first to adopt an ecological framework^(10)^ to examine if individual factors, social networks and neighbourhood environments are linked to changes in the dietary intake of a diverse population of adults during an extended crisis: the COVID-19 pandemic. These insights are needed to understand the complex causes of health disparities and how these are exacerbated during a public health emergency, especially among low-income individuals who face multiple levels of barriers to health. Another major strength is the use of a large and longitudinal panel survey that comprises a sample representative of LA County. LA County is an urban area with diverse socio-demographics, income levels and neighbourhood environments, providing extensive variability that allowed for testing these aims.

### Limitations

Given the size and diversity of LA County, it is plausible that the findings may generalise to other metropolitan areas in the USA. However, these results may not generalise to populations living in more homogenous urban areas or those living in rural areas. Second, these data are based on self-report, which is subject to self-report bias. Additionally, this study focuses on the residential food environment, without considering the food environments where participants work, commute or do other daily activities that have been found to be a more important source of influence on food choice^(46)^. Finally, this study utilises FFQ, which have some notable limitations^(47)^, despite being one of the more predominantly used tools to assess dietary intake.

### Implications

This study focused on adults living in LA County, a diverse area in terms of socio-demographics and social and neighbourhood environments, during the peak of the COVID-19 pandemic. These findings emphasise the complexity of this system-wide crisis and the vulnerability and volatility of health outcomes for groups who experience risk factors at multiple socio-ecological levels. Historically, the diet disparities literature has focused on simple ‘cause and effect’ relationships between a few factors, within one isolated level of influence^(10)^. Yet, there may be interactive relationships between multilevel factors, as was emphasised by the findings of this study. As such, studies that comprehensively consider these complex, multilevel factors are crucial, especially during unprecedented, system-wide changes such as those resulting from the COVID-19 pandemic.

Overall, this study pinpoints key populations (e.g. individuals with less than a college degree, low-income groups with constrained social networks) who should be the focus of multilevel dietary interventions. Though the USA is currently transitioning out of the COVID-19 pandemic into an endemic phase, the social and economic repercussions of the pandemic are still widespread, and the related health burdens of underserved groups are likely to persist. Furthermore, the populations identified in this study will likely continue to be vulnerable to the health effects of future system-wide crises, including natural disasters, disease outbreaks or significant economic downturns^(48)^. Thus, widespread interventions and improvement in policy are still needed, as has been comprehensively discussed elsewhere^(49,50)^. For example, following the lead of other developed countries, policies that reduce inequalities in socio-economic and social barriers to health would be beneficial. A few well-supported policies include increased funding for schools in low-income areas to reduce inequalities in education, subsidised or free childcare programmes for low-income families, expansion of financial and food assistance programmes (e.g. in the USA, programmes like Women, Infants, and Children, the Supplemental Nutrition Assistance Program, and the Section 8 Housing Choice Voucher Program are long overdue for budget increases) and universal healthcare or universal access to affordable and comprehensive health insurance. Until widespread policies that address key groups’ vulnerabilities to intersecting health and socio-economic shocks are in place, these groups will continue to suffer amid future global crises^(49,50)^.

## References

[1] United States Department of Agriculture (2021) Dietary guidelines for Americans. https://www.fns.usda.gov/cnpp/dietary-guidelines-americans (accessed May 2021).

[2] Lee-Kwan SH , Moore LV , Blanck HM et al. (2017) Disparities in state-specific adult fruit and vegetable consumption—United States, 2015. MMWR Morb Mortal Wkly Rep 66, 1241. 29145355 10.15585/mmwr.mm6645a1 PMC5726245

[3] Rosinger A , Herrick K , Gahche J et al. (2017) Sugar-sweetened beverage consumption among U.S. adults, 2011–2014. NCHS Data Brief (279), 1–8. 28135185

[4] The US Burden of Disease Collaborators (2018) The state of US health, 1990–2016: burden of diseases, injuries, and risk factors among US states. JAMA 319, 1444–1472. 29634829 10.1001/jama.2018.0158 PMC5933332

[5] Hales CM , Carroll MD , Fryar CD et al. (2020) Prevalence of obesity and severe obesity among adults: United States, 2017–2018. NCHS Data Brief 360, 1–8. 32487284

[6] González-Monroy C , Gómez-Gómez I , Olarte-Sánchez CM et al. (2021) Eating behaviour changes during the COVID-19 pandemic: a systematic review of longitudinal studies. Int J Environ Res Public Health 18, 11130. 34769648 10.3390/ijerph182111130 PMC8582896

[7] Bennett G , Young E , Butler I et al. (2021) The impact of lockdown during the COVID-19 outbreak on dietary habits in various population groups: a scoping review. Front Nutr 8, 626432. 33748175 10.3389/fnut.2021.626432 PMC7969646

[8] Mekanna AN , Panchal SK & Li L (2023) Beyond lockdowns: a systematic review of the impacts of COVID-19 lockdowns on dietary pattern, physical activity, body weight, and food security. Nutr Rev 81, 790–803. 36250794 10.1093/nutrit/nuac088 PMC9619764

[9] Haspel T (2023) Nutrition research has led us astray. Here’s what we should study instead. The Washington Post. https://www.washingtonpost.com/lifestyle/food/nutrition-research-has-led-us-astray-heres-what-we-should-study-instead/2020/07/23/b229257a-cc58-11ea-91f1-28aca4d833a0_story.html (accessed March 2024).

[10] Story M , Kaphingst KM , Robinson-O’Brien R et al. (2008) Creating healthy food and eating environments: policy and environmental approaches. Annu Rev Public Health 29, 253–272. 18031223 10.1146/annurev.publhealth.29.020907.090926

[11] de la Haye K , Wilson J , Bruine de Bruin W et al. (2021) Enough to Eat: The Impact of COVID-19 on Food Insecurity and the Food Environment in LA County April 2020–September 2021. Los Angeles County and University of Southern California. https://publicexchange.usc.edu/wp-content/uploads/2021/10/Enough-to-Eat.pdf (accessed March 2023).

[12] Gostin LO & Wiley LF (2020) Governmental public health powers during the COVID-19 pandemic: stay-at-home orders, business closures, and travel restrictions. JAMA 323, 2137–2138. 32239184 10.1001/jama.2020.5460

[13] Wernli D , Clausin M , Antulov-Fantulin N et al. (2021) Building a multisystemic understanding of societal resilience to the COVID-19 pandemic. BMJ Glob Health 6, e006794. 10.1136/bmjgh-2021-006794 PMC8300552 34301677

[14] Völker B (2023) Networks in lockdown: the consequences of COVID-19 for social relationships and feelings of loneliness. Soc Netw 72, 1–12. 10.1016/j.socnet.2022.08.001 PMC9359936 35968494

[15] Gauthier GR , Smith JA , García C et al. (2021) Exacerbating Inequalities: social networks, racial/ethnic disparities, and the COVID-19 pandemic in the United States. J Gerontol Ser B 76, e88–e92. 10.1093/geronb/gbaa117 PMC7454830 32756978

[16] Miller S , Bruine de Bruin W , Livings M et al. (2021) Self-reported dietary changes among Los Angeles County adults during the COVID-19 pandemic. Appetite 166, 105586. 34217761 10.1016/j.appet.2021.105586 PMC9756093

[17] Lin P , Hillstrom K , Gottesman K et al. (2023) Financial and other life stressors, psychological distress, and food and beverage consumption among students attending a large California State University during the COVID-19 pandemic. Int J Environ Res Public Health 20, 3668. 36834363 10.3390/ijerph20043668 PMC9965632

[18] Byker Shanks C , Houghtaling B , Shanks J et al. (2022) Disparities in dietary practices during the COVID-19 pandemic by food security status. Prev Med Rep 28, 101830. 35601457 10.1016/j.pmedr.2022.101830 PMC9113950

[19] Park S , Lee SH , Yaroch AL et al. (2022) Reported changes in eating habits related to less healthy foods and beverages during the COVID-19 pandemic among US adults. Nutrients 14, 526. 35276885 10.3390/nu14030526 PMC8838827

[20] Park S , Lee SH & Blanck HM (2023) Characteristics associated with being a high consumer of sweet foods and sugar-sweetened beverages among US adults during the COVID-19 pandemic, 2021. Nutrients 15, 2363. 37242246 10.3390/nu15102363 PMC10222205

[21] LA County Department of Public Health (2023) LA County Daily COVID-19 Data. http://www.publichealth.lacounty.gov/media/Coronavirus/data/index.html (accessed December 2023).

[22] University of Southern California (2020) Understanding America Study. https://uasdata.usc.edu/index.php (accessed November 2020).

[23] UCLA Center for Health Policy Research (2021) Design and Methods. https://healthpolicy.ucla.edu/chis/design/Pages/questionnairesEnglish.aspx (accessed November 2021).

[24] Bruine de Bruin W (2021) Age differences in COVID-19 risk perceptions and mental health: evidence from a national US survey conducted in March 2020. J Gerontol Ser B 76, e24–e29. 10.1093/geronb/gbaa074 PMC7542924 32470120

[25] Kroenke K , Spitzer RL , Williams JB et al. (2009) An ultra-brief screening scale for anxiety and depression: the PHQ–4. Psychosomatics 50, 613–621. 19996233 10.1176/appi.psy.50.6.613

[26] Cafiero C , Viviani S & Nord M (2018) Food security measurement in a global context: the food insecurity experience scale. Meas 116, 146–152.

[27] Bourdieu P (2018) The Forms of Capital. Abingdon, United Kingdom: Routledge.

[28] Bruine de Bruin W , Parker AM , Galesic M et al. (2019) Reports of social circles’ and own vaccination behavior: a national longitudinal survey. Health Psychol 38, 975. 31259597 10.1037/hea0000771 PMC7038818

[29] Galesic M , Bruine de Bruin W , Dumas M et al. (2018) Asking about social circles improves election predictions. Nat Hum Behav 2, 187–193.

[30] Cobb LK , Appel LJ , Franco M et al. (2015) The relationship of the local food environment with obesity: a systematic review of methods, study quality, and results. Obesity 23, 1331–1344. 26096983 10.1002/oby.21118 PMC4482774

[31] Morland KB & Evenson KR (2009) Obesity prevalence and the local food environment. Health Place 15, 491–495. 19022700 10.1016/j.healthplace.2008.09.004 PMC4964264

[32] Drewnowski A , Aggarwal A , Hurvitz PM et al. (2012) Obesity and supermarket access: proximity or price? Am J Public Health 102, e74–e80. 22698052 10.2105/AJPH.2012.300660 PMC3464835

[33] Engle S , Stromme J & Zhou A (2020) Staying at home: mobility effects of COVID-19. Working paper available at SSRN: =https://ssrn.com/abstract=3565703 or 10.2139/ssrn.3565703.

[34] United States Department of Agriculture (2021) Food Access Research Atlas. https://www.ers.usda.gov/data-products/food-access-research-atlas (accessed December 2021).

[35] California Health and Human Services (2021) Modified Retail Food Environment Index. https://data.chhs.ca.gov/dataset/modified-retail-food-environment-index (accessed November 2021).

[36] Wild LE , Walters M , Powell A et al. (2022) County-level social vulnerability is positively associated with cardiometabolic disease in Colorado. Int J Environ Res Public Health 19, 2202. 35206386 10.3390/ijerph19042202 PMC8872484

[37] Yu C-Y , Woo A , Emrich CT et al. (2020) Social Vulnerability Index and obesity: an empirical study in the US. Cities 97, 102531.

[38] Center for Disease Control (2022) Social Vulnerability Index (SVI). https://www.atsdr.cdc.gov/placeandhealth/svi/index.html (accessed October 2022).

[39] Moreno C , Wykes T , Galderisi S et al. (2020) How mental health care should change as a consequence of the COVID-19 pandemic. Lancet Psychiatry 7, 813–824. 32682460 10.1016/S2215-0366(20)30307-2 PMC7365642

[40] Wang Y & Beydoun MA (2007) The obesity epidemic in the United States—gender, age, socioeconomic, racial/ethnic, and geographic characteristics: a systematic review and meta-regression analysis. Epidemiol Rev 29, 6–28. 17510091 10.1093/epirev/mxm007

[41] Cohen S , Doyle WJ , Skoner DP et al. (1997) Social ties and susceptibility to the common cold. JAMA 277, 1940–1944. 9200634

[42] Vogt TM , Mullooly JP , Ernst D et al. (1992) Social networks as predictors of ischemic heart disease, cancer, stroke and hypertension: incidence, survival and mortality. J Clin Epidemiol 45, 659–666. 1607905 10.1016/0895-4356(92)90138-d

[43] Walker RE , Keane CR & Burke JG (2010) Disparities and access to healthy food in the United States: a review of food deserts literature. Health Place 16, 876–884. 20462784 10.1016/j.healthplace.2010.04.013

[44] Cohen S & Wills TA (1985) Stress, social support, and the buffering hypothesis. Psychol Bull 98, 310. 3901065

[45] Domínguez S & Arford T (2010) It is all about who you know: Social capital and health in low-income communities. Health Sociol Rev 19, 114–129.

[46] García Bulle Bueno B, Horn AL, Bell BM, et al. (2024) Effect of mobile food environments on fast food visits. Nature Communications 15(1), 2291. 10.1038/s41467-024-46425-2 PMC10937966 38480685

[47] Kristal AR , Peters U & Potter JD (2005) Is it time to abandon the food frequency questionnaire? Cancer Epidemiol Prev Biomark 14, 2826–2828. 10.1158/1055-9965.EPI-12-ED1 16364996

[48] Arthi V & Parman J (2021) Disease, downturns, and wellbeing: economic history and the long-run impacts of COVID-19. Explor Econ Hist 79, 101381. 33162564 10.1016/j.eeh.2020.101381 PMC7606070

[49] Bleich SN , Jarlenski MP , Bell CN et al. (2012) Health inequalities: trends, progress, and policy. Ann Rev Public Health 33, 7–40. 22224876 10.1146/annurev-publhealth-031811-124658 PMC3745020

[50] Basterra EL , Naidoo M , Calvacanti D et al. (2023) Social protection in global crises: a gap between evidence and action. BMJ Global Health 8, e013980. 10.1136/bmjgh-2023-013980 PMC10626864 37923321

